# Intronic RNAs constitute the major fraction of the non-coding RNA in mammalian cells

**DOI:** 10.1186/1471-2164-13-504

**Published:** 2012-09-24

**Authors:** Georges St Laurent, Dmitry Shtokalo, Michael R Tackett, Zhaoqing Yang, Tatyana Eremina, Claes Wahlestedt, Silvio Urcuqui-Inchima, Bernd Seilheimer, Timothy A McCaffrey, Philipp Kapranov

**Affiliations:** 1 Immunovirology – Biogenisis Group, University of Antioquia, Medellin, A.A. 1226, Colombia; 2 St. Laurent Institute, One Kendall Square, Cambridge, MA, USA; 3 A.P.Ershov Institute of Informatics Systems SB RAS, 6, Acad. Lavrentjev pr, Novosibirsk, 630090, Russia; 4 University of Miami Miller School of Medicine, 1501 NW 10th Ave, Miami, FL, 33136, USA; 5 Biologische Heilmittel Heel GmbH, Dr.-Reckeweg-Str. 2-4, Baden-Baden, 76532, Germany; 6 Department of Medicine, Division of Genomic Medicine, The George Washington University Medical Center, 2300 I St. NW, Washington, DC, USA

## Abstract

**Background:**

The function of RNA from the non-coding (the so called “dark matter”) regions of the genome has been a subject of considerable recent debate. Perhaps the most controversy is regarding the function of RNAs found in introns of annotated transcripts, where most of the reads that map outside of exons are usually found. However, it has been reported that the levels of RNA in introns are minor relative to those of the corresponding exons, and that changes in the levels of intronic RNAs correlate tightly with that of adjacent exons. This would suggest that RNAs produced from the vast expanse of intronic space are just pieces of pre-mRNAs or excised introns *en route* to degradation.

**Results:**

We present data that challenges the notion that intronic RNAs are mere by-standers in the cell. By performing a highly quantitative RNAseq analysis of transcriptome changes during an inflammation time course, we show that intronic RNAs have a number of features that would be expected from functional, standalone RNA species. We show that there are thousands of introns in the mouse genome that generate RNAs whose overall abundance, which changes throughout the inflammation timecourse, and other properties suggest that they function in yet unknown ways.

**Conclusions:**

So far, the focus of non-coding RNA discovery has shied away from intronic regions as those were believed to simply encode parts of pre-mRNAs. Results presented here suggest a very different situation – the sequences encoded in the introns appear to harbor a yet unexplored reservoir of novel, functional RNAs. As such, they should not be ignored in surveys of functional transcripts or other genomic studies.

## Background

Mammalian cells require molecular machineries with sufficient complexity and diversity to acquire, process, and distribute vast amounts of information. The unique features of non-coding RNAs could facilitate key steps in the information processing activities of hundreds of regulatory pathways, suggesting a role for them as the major informational “currency” of the cell [1,2]. The large-scale efforts that discovered pervasive transcription in mammalian genomes determined that many such transcripts came from completely un-annotated intergenic and intronic regions. Often referred to as “dark matter” [3], these non-coding regions outside of annotated exons produce an impressive array of transcriptional products that undergo intricate and complex processing in diverse pathways [4,5]. While the existence and function of RNAs originating from this large space of non-coding regions of the genome has been until recently a subject of considerable debate [6-9], improved RNA-seq methods have now established their existence [10] and the recent reports from the ENCODE consortium leave little doubt as to their prevalence [11,12]. In fact, single molecule RNA-seq recently demonstrated that the majority of the mass of a human cellular pool of RNA comes from non-exonic transcription [10], outweighing protein coding mRNAs, and underscoring the potential importance of this class of transcriptional output.

However, the prevalent belief is that these RNAs simply represent pre-mRNAs en route to splicing or spliced-out introns en route to degradation. Moreover, it has been suggested that the levels of RNAs in the intronic regions are consistently lower than those in exons and that the levels of intronic and exonic RNAs highly correlate [9] – all consistent with a simple notion that pre-mRNAs or splicing by-products comprise most of the intronic signal, and arguing against any broad, potentially functional, stand-alone RNA molecules encoded in these regions.

To gain insight into the functional significance of non-exonic RNA, we asked whether introns could indeed harbor functional transcripts in the cell whose features and physiological behavior differed from that of pre-mRNAs or spliced out introns. In this report, we present the results of a global investigation of intronic transcription during the mammalian inflammation cascade. The cascade sets in motion a series of intricate responses to the perturbation of invading pathogens, using many interlocking feedback loops to determine the precise nature and extent of the challenge, and carefully regulating the corresponding pathways to resolution. Thus, it is expected to require a significant intracellular information exchange, where we and others expect non-coding RNAs to function [13-15] and as such provides a relevant biological model to study the latter. Here we use single molecule RNA-seq methods, and bioinformatic analysis adapted to accurately capture RNAs transcribed from non-exonic regions. We present data that challenges the notion that intronic RNAs are mere by-standers in the cell, but rather suggest these they give rise to RNAs that persist, change in response to inflammation, and are regulated differently from their exonic counterparts.

## Results

### Intronic RNAs constitute the major component of the mammalian transcriptome

Mice were treated with lipo-polysaccharide (LPS) by inhalation, followed by isolation of RNA from lung at 3, 6, 12, 24 and 48 hours post-treatment. In total 42 animals were studied: 7 animals at each time-point and a group of control, un-treated animals. We have chosen the single-molecule sequencing (SMS) platform for RNA analysis due to its superior performance and reproducibility for quantification of RNA expression, in part due to the fact that it does not depend on PCR amplification and ligation for the library preparation [16]. Total RNA rather than the polyA + fraction was used because the latter lacks a significant proportion of the complexity of RNA present in the cell [10,17].

Total RNA from each sample was treated with DNAse, subjected to a RiboMinus procedure to deplete ribosomal RNAs and sequenced by SMS. The basic statistics reflecting the distribution of mapped reads in annotated exons, introns and intergenic regions are shown in Table 1. On average, we generated 21.1-28.8 M mapped SMS reads per animal per time point, of which 12.2–16.6 M could be mapped uniquely to the genome. After subtracting reads mapping to ribosomal RNAs, mitochondria and genomic ribosomal RNA repeats from total uniquely-mapping reads, each sample was found to yield on average 7.0–9.6 M informative reads, which serve as the basis for all subsequent analyses in this work (Table 1). The LPS signal transduction cascade rapidly triggers changes in transcriptional regulation, resulting in the induction and repression of hundreds of genes in many cell types [18-22]. Detection of these well-known inflammation markers serves as an indicator of the performance of the experimental system. As expected, we detected induction of over 1000 genes as early as the 3 hr time point, including the major components of the mammalian inflammatory pathways, such as chemokine ligands and their receptors, interleukins and their receptors, growth factors, complement components and others (St. Laurent et al., manuscript in preparation).

**Table 1:** Distribution of mapped reads among different genomic annotations

**Time point (hours)**	**All mapped reads**	**Uniquely mapped reads**	**Uniquely mapped reads that are rRNA or rRNA repeat**	**Uniquely mapped reads that are chrM**	**Informative reads**	Informative reads that overlap with exons*	**% of non-exonic reads**	**Informative reads that overlap with introns**	**% of Informative reads that overlap with introns**	**% of Informative reads that overlap with introns as a fraction of non-exonic reads**	**Informative reads that are intergenic**	**% of Informative reads that are intergenic**
0	172,810,082	100,490,763	37,454,298	4,040,580	58,995,885	21,353,240	63.8 %	25,213,131	42.7 %	67.0 %	12,429,515	21.1 %
3	188,573,448	111,280,605	39,613,334	4,490,786	67,176,485	24,307,053	63.8 %	28,235,047	42.0 %	65.9 %	14,634,386	21.8 %
6	201,295,896	116,218,502	47,449,481	4,055,092	64,713,929	22,823,246	64.7 %	28,228,817	43.6 %	67.4 %	13,661,866	21.1 %
12	170,486,061	93,829,203	39,479,285	3,924,386	50,425,532	18,187,717	63.9 %	21,318,414	42.3 %	66.1 %	10,919,401	21.7 %
24	157,234,367	91,301,394	34,072,429	4,477,595	52,751,370	18,760,773	64.4 %	22,667,538	43.0 %	66.7 %	11,323,060	21.5 %
48	147,848,170	85,053,947	32,412,171	3,634,177	49,007,599	17,832,101	63.6 %	20,929,521	42.7 %	67.1 %	10,245,978	20.9 %

*Exons were defined by the UCSC Genes track (Methods).

Consistent with our previously published-results [10], 63.6–64.7% of the informative reads mapped outside exons of annotated genes as defined by the UCSC Genes track [23] and thus represent the RNAs from the non-coding portion of the genome (Table 1). The proportion of the latter was fairly consistent across all time points (Table 1) and all animals (data not shown). The relative mass of intronic RNAs dominated the non-exonic RNA population: ~ 66% of non-exonic informative reads fell within introns (Table 1). This amounted to ~ 42% of all informative reads – a higher fraction than that of reads mapping to annotated exons (~36%, Table 1). This observation posed an important question: whether the intronic RNAs simply represented unspliced pre-mRNAs or whether they could indeed be a source of stand-alone, functional RNAs. If the former is true, then the fraction of the so-called “dark matter” RNA in the cell is fairly small and could be explained by the exons of yet un-annotated intergenic RNAs. On the other hand, if the latter is true, it would mean that intronic RNAs harbor a significant amount of functional RNAs.

**Figure 1:**
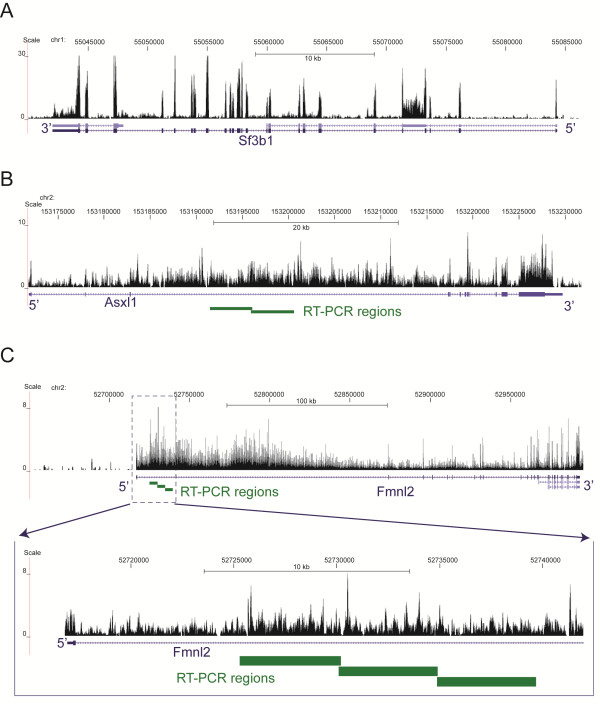
**RNAseq profiles representing annotations with little intronic signal (A) and extensive intronic signal (B & C).** The profiles are based on the control RNA samples. Positions of RT-PCR products presented in Figure 7 are shown (see Additional file 4: Table S3 for more details).

It is worth noting that in our analysis intronic RNAs refer to any RNAs encoded by genomic regions annotated as introns, including excised introns, alternative isoforms of exons, as well as standalone sense or antisense transcripts transcribed from their own promoters that overlap intronic regions. The first clue that intronic RNAs should not be disregarded came from the observation that the signal from intronic RNA was not evenly spread among genes: rather, annotated genes differed significantly in the amounts of intronic RNAs (Figure 1). On one side, Sf3b1 represents an example of a gene with high RNAseq signal in exons and very low signal in most introns, mirroring the two annotated exon-intron structures of the gene (Figure 1A). In contrast, the RNAseq signal in the Asxl1 and Fmnl2 genes reached comparable levels in both exons and introns, (Figure 1B and C). The presence of transcripts like Sf3b1 suggests that relatively high levels of intronic RNAs found in Asxl1 and Fmnl2 genes is not a general property of mouse genes. To calculate how many transcripts resembled Sf3bl in terms of the low level of intronic RNAs, we compared the RNAseq densities in the introns to that of exons in each annotated mouse transcript. Since it is not uncommon even in transcripts with overall low intronic RNAseq signal to have some introns with high signal, as exemplified by a retained intron of Sf3b1 (Figure 1), we used the entire lengths of intronic and exonic portion of each transcript for calculation of intronic and exonic densities in this analysis. We calculated average intronic and exonic densities for each of the 6 timepoints by averaging across 7 animals in each timepoint. We then sorted the transcripts by minimum exonic density and picked the top half in order to remove transcripts with low read counts in exons that would result in imprecise calculations. We then calculated the minimum intron/exon ratios across the 6 datapoints for each transcript and selected transcripts whose corresponding ratios were ≤0.1 that would be similar to that exhibited by Sf3b1 (0.062). We selected the minimal intron/exon ratio because the levels of intronic RNAs were subject to change throughout the time course often without the concomitant change in exons, and as such the minimum intron/exon ratio would more accurately represent the baseline intronic RNA level relative to its exons for a given locus. In total 7,779 (32.6%) of expressed annotated mouse transcripts used in this analysis satisfied this very stringent criterion. This means that for thousands of different mature transcripts, the intronic RNAs are efficiently removed by the cell. One would expect that intronic RNAs that escape removal and persist within this cellular context could play some functional role.

### Levels of intronic RNAs are independent of those of exons or other introns

**Figure 2:**
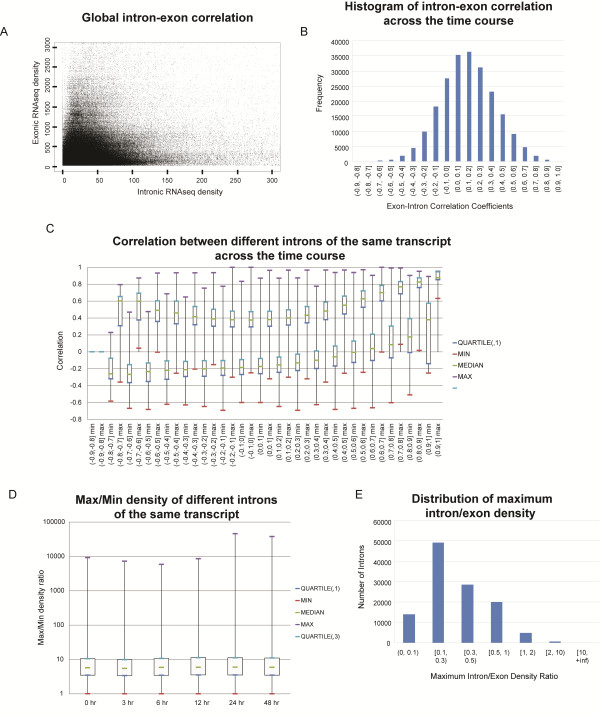
**Correlation between levels of exonic and intronic RNAs.** (**A**) Plot of normalized read densities for every intron and corresponding exons: the exon-intron densities for every animal were combined and the top half of the dataset based the highest exonic densities is plotted. (**B**) Histogram of Spearman rank correlations obtained for every intron-exon pairs throughout the time course of LPS treatment. (**C**) Histogram of minimal (min) and maximum (max) correlations (Y-axis) between an intron and all other introns of the same transcript. The X-axis shows the correlation of the intron with the corresponding exons of the transcript as shown in the panel B. (**D**) Boxplots of the ratio of maximum/minimum intronic RNAseq density of different introns in the same transcript for each time point. Intronic density was calculated as the average of the 7 animals per each time point. (**E**) Histogram of maximum ratio of intron/exon densities for each intron that could be found in any of the 42 animals at any timepoint.

As the next step, we investigated whether levels of intronic RNAs in general mirrored those of the exons of their associated transcripts, or whether the two were relatively independent from each other. Some controversy in this regard exists in the literature. The report by van Bakel et. al. [9] found a near perfect correlation between the levels of corresponding intronic and exonic RNAs as shown in Figure 1C of that report. However, our previous work has suggested significant levels of variation between the RNA levels of introns and exons [10,17]. The discrepancy could be explained by the fact that the van Bakel et. al. work has relied on PCR amplification during their library preparation, which can distort levels of original RNA populations [17,24,25], while SMS used in our previous and current work avoids amplification altogether, and provides a sensitive, linear, and highly reproducible signal for RNAseq analysis. We calculated the global coefficient of correlation between the densities of reads in introns and exons of each annotated gene in each sample. Since we have previously observed that different introns of the same gene can have very different densities of RNAseq signal [10] (also see below), for this analysis and all subsequent analyses we treated each intron separately by comparing it to the density of exonic reads for the entire mature transcript that harbors it. An intron with the same genomic coordinates found in more than one annotated transcript structure was compared to each such transcript separately (Methods, also Additional file 1: Figure S1). The distribution plot of 8,918,127 total data points of exon-intron densities (Methods) revealed significant spread with no general trend (Figure 2A). Consistently, Spearman rank correlation performed globally on this data set measured 0.4. Overall, this suggests that on a genome-wide basis the density of RNA signal for any given intron exhibits a wide divergence from that for the exons of the corresponding transcript.

We then calculated Spearman rank correlation between RNAseq densities of each intron and the corresponding exons throughout the time course by comparing 42 exon-intron densities for each animal in each time point for each intron-exon pair (Methods). The histogram of the resulting correlation coefficients for 220,645 exon-intron pairs from this analysis is shown in Figure 2B. Interestingly, 62,848 (28.5%) pairs showed negative or zero correlation coefficients and, an additional 71,591 (32.4%) pairs have positive, but weak (0 < 0.2) correlations. Only 16,316 (7.4%) pairs showed relatively strong correlation (>0.5).

We then asked how well different introns of the same transcript correlate with each other. We used two metrics – correlation of different introns of the same transcript with each other throughout the time course, and the range of differences of the intronic RNAseq signal within the same transcript. We expected that if an intron indeed harbored independently regulated transcripts, these transcripts would not correlate with the levels of either exons or other introns from the same locus. The correlations between an intron N and all other introns in a given locus were split into bins according to the correlation that this intron had with the corresponding exons as shown in Figure 2B. Finally, for each intron we plotted maximum and minimum correlation values with other introns of the same locus (Figure 2C).

Indeed, introns that correlated well with their corresponding exons also had higher correlations with the other introns, as evidenced by the upward trend of the minimal correlation box plots in the right portion of the Figure 2C. This would suggest that they most likely represent pre-mRNA, at least in this biological source. On the other hand, introns that did not correlate well with exons also had a tendency to correlate less with other introns of the same transcript as evidenced by the decreased minimal correlation on Figure 2C. The contrasting statistical behavior is consistent with these introns harboring independently-regulated transcripts.

We then asked how introns of the same transcript might differ in terms of abundance with their corresponding RNAs. For each transcript with 2 or more introns analyzed in Figure 2C above, we calculated the average density of each intron per each time point (average of 7 animals). We then calculated the ratios of the introns with the maximum and the minimum densities of each transcript - the results are shown in Figure 2D. Interestingly, the median maximum/minimum density ratio was in the range of 5.5-6.0, showing that levels of intronic RNAs within the same transcript could indeed vary significantly. Again, this is consistent with separate fates of RNA made from different introns even from the same transcript.

### Introns encode relatively abundant RNA species

The median ratio of intronic densities to their corresponding exonic densities was found to be 11.95% across all samples in the “Total Intron-Exon Pair-Wise Dataset” (Methods). However, as mentioned above we also observed heterogeneity in the level of intronic RNA signal, even among different introns of the same gene. Therefore, we asked how many individual introns have at least comparable or higher read densities than that of their corresponding exons in at least one animal at any time point. We calculated the maximum ratio of intron/exon densities for each intron that could be found in any of the 42 animals at any timepoint. The distribution of these ratios is shown in Figure 2E. In total, out of 117,314 introns with unique start and stop genomic coordinates tested here, 25,821 had at least 50% of the density of the corresponding exons and 5,753 had higher densities than the exons in at least one animal in at least one time point (Figure 2E).

We were intrigued by the introns whose densities are higher than that of exons in this analysis and investigated this further. The introns were stratified based on the maximum ratio of intron/exon density. In the >10 fold category, represented by 96 introns, most (~90%) contained annotated non-coding RNAs, with half of the introns containing snoRNAs found only in the Ensembl database (Table 2). While the reads corresponding to snoRNAs annotated by UCSC were removed from this analysis, those corresponding to snoRNAs specific to the Ensembl database were not. In some cases, the high level of signal also extended outside of the boundary of the UCSC snoRNAs, giving the entire intron a high average RNAseq density (Table 2). This overall result was not un-expected considering that the most abundant intronic RNAs are the ones more likely found using the standard molecular biological techniques and thus would be annotated in the genomic databases. Detection of annotated non-coding RNAs in the top tier introns validated one of the criteria used to identify this tier, specifically the maximum intron/exon density ratio (also see below for more details).

**Table 2:** Annotation of abundant introns based on presence of known small RNAs

	**Maximum intron/Exon density**			
	**>10 fold**	**2-10 fold**	**1-2 fold**	**1 fold and above**
Total introns*	96	707	4,950	5,753
UCSC snoRNAs	34	19	23	76
Ensemble snoRNAs	48	31	17	96
Non-mouse snoRNAs	1	0	0	1
Ensemble snRNAs	2	7	8	17
RNA repeats	3	14	52	69
Un-annotated	8	636	4,850	5,494
Un-annotated %	8.3%	90.0%	98.0%	95.5%

*Introns overlapping an annotation were removed from the subsequent analysis. For example, introns overlapping UCSC snoRNAs were not to be used for comparison with Ensembl snoRNAs and so on.

Also as expected, once we lowered the maximum intron/exon density ratio to include the introns with still significant, but lower ratio of 2–10 and 1–2 fold, the situation reversed. More than 90% of introns in these much more numerous categories did not have any annotated RNA (Table 2). However, the overall trend remained the same: the fraction of annotated RNAs in the 2–10 fold introns is higher than in the 1–2 fold ones. Overall, out of 5,753 introns whose density was equal or higher than that of the corresponding exons in at least one animal, 5,494 (95.5%) did not have annotated RNAs (Table 2).

**Figure 3:**
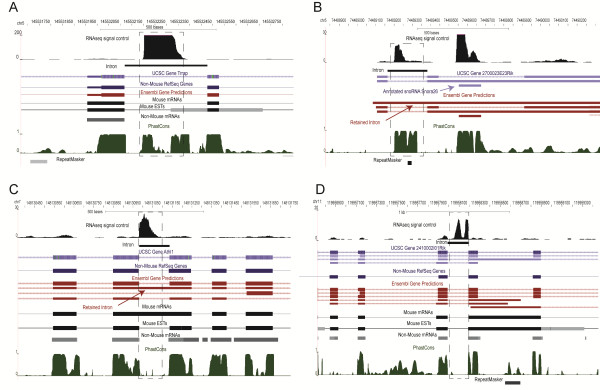
**Examples of novel intronic RNAs whose RNAseq densities in the control (untreated with LPS) sample are >10 fold higher than those of the corresponding exons.** The PhastCons track from the UCSC browser represents the Euarchontoglire subset. One of these introns is close to an annotated snoRNA Snora26 (B), which is interesting considering that sometimes multiple snoRNAs are encoded within the same locus.

Interestingly, even in the >10 fold group, 8 introns appear to harbor un-annotated RNAs. One of these 8 was determined to be a snoRNA found previously in platypus based on the BLAST alignment of the sequence, but not annotated in any database used above (not shown). The remaining 7 seem to represent novel RNAs with no BLAST hits to any sequence of known function and 4 examples are shown in Figure 3. They are likely to be functional considering that they come from a class of introns heavily enriched in annotated functional non-coding RNAs, but some of them are conserved (Figure 3A and B) and some are not (Figure 3C and D) as judged by their PhastCons scores. This is consistent with the complex relationship between non-coding RNAs and sequence conservation [26].

These two examples shown in Figure 3B and C are of additional interest: they are annotated as “retained” in a long partially spliced RNA by Ensembl. However, the profile of the RNAseq signal which is much higher in each of the two introns than in the surrounding exons suggests that they function on their own and not as part of a longer mRNA as suggested by the annotation. This exemplifies a larger theme which stipulates that an intron could function as a separate entity even if it is currently annotated as part of a larger RNA species.

### Levels of intronic RNA can have the same biological variance as their corresponding exons

Accurate and precise regulation of a biological molecule separates functional behavior from noise. Therefore, we asked whether the steady-state levels of intronic RNAs could be as tightly regulated as those of exons. We reasoned that the level of control exerted over the steady-state level of a transcript would manifest itself in the variation of its levels measured among different genetically-identical individuals maintained in the same conditions. Levels of tightly regulated RNAs would vary less than those of RNAs under little regulatory control. Our system is well-suited for measurement of such variation – we have profiled 7 animals of the same gender and similar age per time point from a genetically-homogeneous strain of mice using SMS, which generates highly reproducible results [17], a key to the endeavor.

To accomplish this, we calculated the coefficient of variation (CV) of each individual intron and the corresponding exons among 7 animals for each time point, resulting in 1,231,535 intron-exon combinations (Methods). While the much lower intronic density precludes a direct comparison between the CV’s of the introns and exons (Methods), we asked how many introns had the same or lower CV than the corresponding exons at any time point. Surprisingly, 40,948 out of 98,190 introns used in this analysis satisfied this criterion, despite the fact that the RNAseq median densities within these introns were 20.78% of that of the corresponding exons. In other words, the levels of 41.7% of introns have the same or lower animal-to-animal variation within the same time point of LPS treatment (or control) as the corresponding exons, even though their relative levels are on average ~5 fold lower, suggesting an extraordinary level of biological control of steady state expression of intronic RNA.

### Most of the transcripts changing during LPS inflammation originate from non-coding regions

As a next step, we interrogated the entire genome (exonic, intronic and non-exonic regions) for the presence of RNAs that change during different stages of inflammation irrespective of the presence of annotation. Determining un-annotated RNAs presents a unique challenge because their sequences are not known and the read length is shorter than the sequence of the entire RNA molecule. Therefore, we have chosen an unbiased approach to identify such regions, based on splitting the genome into a series of non-overlapping bins of different sizes, and then counting the number of SMS reads in each bin, in each sample, and then comparing to the control. We have previously reported this approach [17] with the difference that here we used bins of varying sizes for more accurate detection of different types of transcripts (Methods).

**Figure 4:**
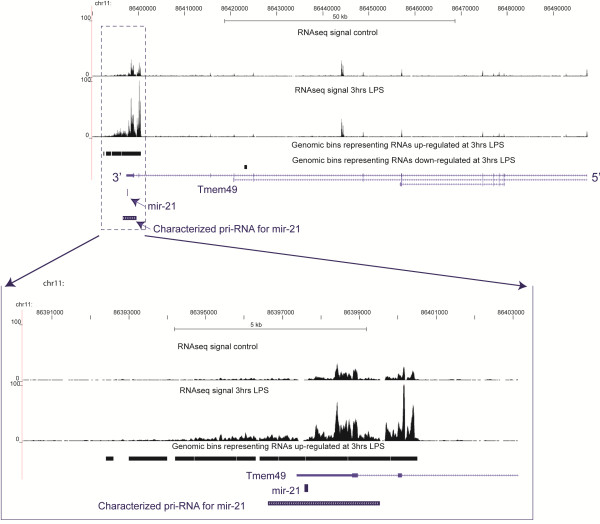
An example of a known non-coding RNA, the primary precursor transcript of mir-21 upregulated at 3 hrs after LPS treatment.

An example of a known non-coding RNA detected with this method, the primary transcript for miRNA mir-21, whose transcription increases during inflammation [27], is shown in Figure 4. A portion of the Tmem49 locus where mir-21 is located, is heavily upregulated after 3 hours of LPS treatment while the rest of the Tmem49 locus does not exhibit change (Figure 4). The upregulated region also overlaps the primary transcript for miRNA mir-21 characterized in humans [28] but not annotated in the human or mouse transcript databases, such as UCSC Genes.

**Figure 5:**
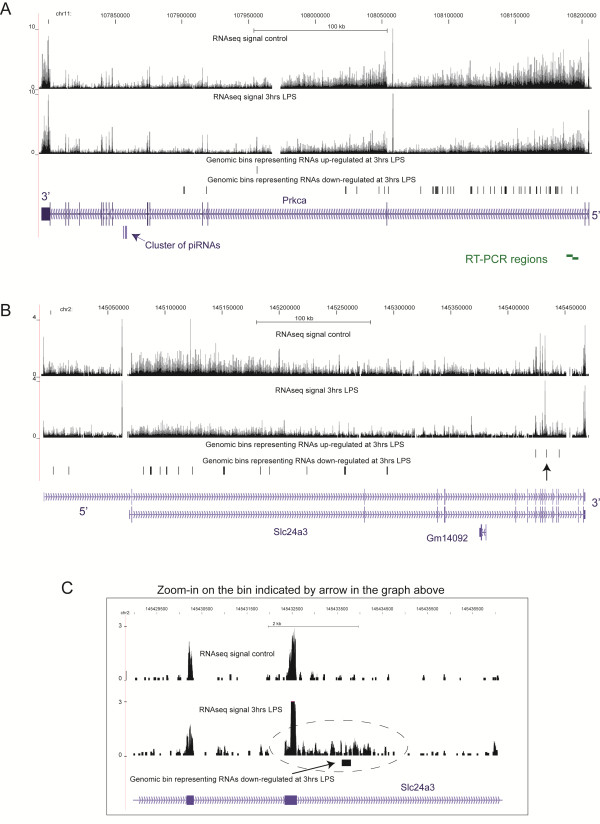
**Examples of intronic RNAs downregulated at 3 hrs LPS with no changes in the exonic RNAs.** The panel (**C**) shows zoom-in around the DE bin marked with an arrow in the panel (**B**). More details in the text. Positions of RT-PCR products presented in Figure 7 are shown (see Additional file 4 Table S3 for more details).

Table 3 summarizes the results of the genome-wide differentially-expressed (DE) bin analysis for all time points. Overall, most changes are seen at the 3 hr time point, followed by the 6 hr time point, consistent with previous reports of expression analysis during LPS induced inflammation [29]. On average, ~80% of all bins that changed at any given time point did not overlap exons of UCSC Genes (Table 3). The majority of DE bins fall within intronic regions: on average, ~70% of all DE bins. Overall, at any given time point, thousands of loci contained at least one DE intronic bin (Table 4). While the trivial explanation of these findings could have been that the intronic regions simply follow the change of the corresponding mature RNAs (the exons), relatively few loci followed this pattern. Interestingly, many intronic transcripts changed without accompanying change in the same direction in the corresponding exons of the gene. Figure 5 shows two such examples: Prkca and Slc24a3, encoding protein kinase C alpha and sodium/potassium/calcium exchanger 3 respectively. Both have a number of DE bins that are down-regulated at 3 hrs distributed throughout the intronic regions of both transcripts and mostly limited to introns with the highest signal, which also happen to be the longest introns of these genes – the second introns (Figure 5). The exonic portions of these transcripts show little change at 3 hrs or any subsequent time point (Additional file 2: Table S1). In fact, the exons of both genes show slight (albeit not statistically significant at p-value = 0.05) change in the opposite direction (upregulation) at 3 hrs (Additional file 2: Table S1). The Spearman rank correlations between the densities for these introns and the exons for both genes throughout the time course were quite low: -0.0936 and 0.0785 respectively (Additional file 2: Table S1). For example, in Prkca, in 4 out of 5 LPS treatment time points, the level of the longest intron was lower than in the controls at statistically-significant levels (p-value < 0.05). However, at no time point were the level of exons different than in the control at the same level of significance (Additional file 2: Table S1). This argues against a simple model where transcription would be shut off for both genes and intronic transcripts would decrease rapidly simply because they are less stable than the mature RNAs. In that case, we would have expected to observe an eventual drop in exonic RNA signal and we did not observe it for either gene during 48 hrs. Furthermore, the average CV for both introns throughout all time points was quite similar to that of the exons (Additional file 2: Table S1), again suggesting that the cells regulate the levels of both these introns as tightly as their corresponding exons, yet quite independently of each other.

**Table 3:** Distribution of differentially expressed (DE) bins among different genomic annotations

	**3 hours**	**6 hours**	**12 hours**	**24 hours**	**48 hours**					
	Down-regulated*	Up-regulated*	Down-regulated*	Up-regulated*	Down-regulated*	Up-regulated*	Down-regulated*	Up-regulated*	Down-regulated*	Up-regulated*
Total number of DE bins	8,316	17,348	4,107	6,746	5,276	5,264	6,714	5,464	6,217	3,939
Exonic bins	392	2,053	249	356	299	434	334	258	203	269
Bins that overlap both exons and introns	648	2,597	409	699	492	704	566	617	429	386
Bins that overlap Ensembl exons	127	297	79	92	79	110	101	104	72	58
Bins that overlap exons of Ensembl retained introns	27	78	29	26	24	20	31	32	25	25
Bins that overlap both Ensembl exons and exons of Ensembl retained introns	3	9	1	1	0	2	5	2	0	1
Intronic bins	6,530	10,274	3,041	5,063	4,062	3,515	5,359	3,995	5,070	2,953
Bins that overlap EST (both exons and introns)	75	223	41	65	57	71	50	53	65	46
Bins that overlap EST (exons only)	25	115	23	34	29	46	19	33	21	16
Bins that overlap EST (introns only)	248	527	73	123	86	96	95	99	91	56
Intergenic bins	241	1,175	162	287	148	266	154	271	241	129
Total number of DE bins	100.00 %	100.00 %	100.00 %	100.00 %	100.00 %	100.00 %	100.00 %	100.00 %	100.00 %	100.00 %
Exonic bins	4.71 %	11.83 %	6.06 %	5.28 %	5.67 %	8.24 %	4.97 %	4.72 %	3.27 %	6.83 %
Bins that overlap both exons and introns	7.79 %	14.97 %	9.96 %	10.36 %	9.33 %	13.37 %	8.43 %	11.29 %	6.90 %	9.80 %
Bins that overlap Ensembl exons	1.53 %	1.71 %	1.92 %	1.36 %	1.50 %	2.09 %	1.50 %	1.90 %	1.16 %	1.47 %
Bins that overlap exons of Ensembl retained introns	0.32 %	0.45 %	0.71 %	0.39 %	0.45 %	0.38 %	0.46 %	0.59 %	0.40 %	0.63 %
Bins that overlap both Ensembl exons and exons of Ensembl retained introns	0.04 %	0.05 %	0.02 %	0.01 %	0.00 %	0.04 %	0.07 %	0.04 %	0.00 %	0.03 %
Intronic bins	78.52 %	59.22 %	74.04 %	75.05 %	76.99 %	66.77 %	79.82 %	73.11 %	81.55 %	74.97 %
Bins that overlap EST (both exons and introns)	0.90 %	1.29 %	1.00 %	0.96 %	1.08 %	1.35 %	0.74 %	0.97 %	1.05 %	1.17 %
Bins that overlap EST (exons only)	0.30 %	0.66 %	0.56 %	0.50 %	0.55 %	0.87 %	0.28 %	0.60 %	0.34 %	0.41 %
Bins that overlap EST (introns only)	2.98 %	3.04 %	1.78 %	1.82 %	1.63 %	1.82 %	1.41 %	1.81 %	1.46 %	1.42 %
Intergenic bins	2.90 %	6.77 %	3.94 %	4.25 %	2.81 %	5.05 %	2.29 %	4.96 %	3.88 %	3.27 %

*Relative to the control time point.

**Table 4:** Distribution of intronic differentially expressed (DE) bins

**All intronic bins:**											
	**3 hours**		**6 hours**		**12 hours**		**24 hours**		**48 hours**		
	Down-regulated*	Up-regulated*	Down-regulated*	Up-regulated*	Down-regulated*	Up-regulated*	Down-regulated*	Up-regulated*	Down-regulated*	Up-regulated*	**Total Up- or Down-Reg. All Time Points**
Intronic bins**	6,530	10,274	3,041	5,063	4,062	3,515	5,359	3,995	5,070	2,953	
Introns overlapping intronic bins	4,331	6,995	3,149	5,152	3,779	3,645	4,063	3,856	4,096	3,087	24,181
Transcripts overlapping intronic bins	5,433	8,463	4,709	7,557	5,387	5,555	5,674	5,522	5,814	4,870	20,061
Loci overlapping intronic bins	2,254	3,622	1,972	3,082	2,315	2,340	2,331	2,321	2,416	2,046	8,016
**Intronic bins with no corresponding changes in the exons:**											
Intronic bins**	3,569	4765	2,292	4,033	3,016	2,665	3,413	3,134	4,131	2,449	
Introns overlapping intronic bins	2,813	4,414	2,435	4,131	2,906	2,849	3,014	3,114	3,514	2,613	19,462
Transcripts overlapping intronic bins	3,890	6,165	3,727	6,098	4,253	4,522	4,422	4,626	5,054	4,186	18,209
Loci overlapping intronic bins	1,556	2,472	1,474	2,316	1,754	1,777	1,713	1,820	1,974	1,620	7,319

*Relative to the control time point; **After removal of bins overlapping exons of Ensembl annotations.

Overall, thousands of such examples exist: just at the 3 hr time point, we found 3,890 annotated transcripts corresponding to 1,556 loci that had at least one intronic down-regulated bin and 6,165/2,472 transcripts/loci that had at least one intronic up-regulated bin with no accompanying changes in exons of these genes (Table 4). Reciprocally, 49.60% to 82.01% of intronic DE bins fell into introns of genes that did not show change at any timepoint (Table 4). This is consistent with the overall low correlation between expression levels of intronic and exonic RNAs (see Figure 2). Multiple such examples existed at each time point as summarized in Table 4. Overall, 7,319 loci had at least one DE intronic bin without accompanying change in the levels of exons in at least one time point.

It is worth noting that even though the dominant trend in the Slc24a3 locus was downregulation of the entire very long intron 2 at the 3 hr timepoint, there is however a portion of intron 11 that is upregulated at the 3 hrs timepoint, and this portion is detected by one DE bin– see Figure 5C. This illustrates the fact that the RNA output is very complex, with different transcripts potentially regulated differently, even when they are derived from the same locus. It also shows that frequently only portions of introns could be retained in stable transcripts and thus a more refined approach like the genomic bins is required.

### Number of introns that potentially contain functional RNAs in one tissue

Perhaps one of the most interesting questions is whether an application of the parameters developed above can enrich for functional non-coding RNAs and if so, how many introns would be detected using such parameters. We have already shown above that the “Maximum Intron/Exon Density Ratio” can enrich for previously-annotated non-coding RNAs (Table 2). We decided to extend this analysis to all parameters developed in this work. To gauge the enrichment for functional non-coding RNAs, we have selected the set of snoRNAs specific to the Ensembl database and not found in the UCSC Genes database used by us as the surrogate for the annotated genome (see above). In this sense, the Ensembl-specific snoRNAs became our test set of known functional non-coding RNAs. We have then asked how many introns would pass any of the parameters used by us above and whether these introns would be enriched in the expressed Ensembl-specific snoRNAs (Methods).

The results of this analysis are summarized in the Table 5. All of the six criteria used gave statistically significant enrichment of the Ensembl-specific snoRNAs (Table 5). The most significant criteria were “Maximum Intron/Exon Density Ratio” and “DE at any time point”. This was followed by the “Coefficient of Variation” and then by ratio of intronic density to that of the intron with the minimal intronic density in the locus (Table 5). The least useful (yet still significant) criteria were “Exon-Intron Correlation” and “Correlation with other introns” that were set to select introns with low correlation with the corresponding exons or other introns of the same locus (Table 5). Overall, 82,481unique introns were found to pass at least one of these parameters, 35,979 passed at least two and 10,111 passed at least three (Table 5). In all cases the detection of the Ensembl-specific snoRNAs was highly significant (Table 5). It is worth emphasizing that introns that passed these criteria are likely to produce functional RNAs in just one tissue (lung) and one condition (inflammation) – other lists will likely exist for other biological systems and these or similar parameters might be helpful in uncovering those. The list of all 82,481 unique introns that were found to pass at least one of these parameters is provided in the Additional file 3: Table S2.

**Table 5:** Number of introns that contain functional RNAs based on various criteria

**Criterion**	**Threshold used**	Number of introns detected *	**Number of ensembl-specific snoRNAs detected**	p-value** of overlap with ensembl-specific snoRNAs	**“Sense” introns (> = 63 % sense reads)**	**“Sense-antisense” introns ([37 %; 63 %) sense reads)**	**“Antisense” introns (<37 % sense reads)**	**Fraction of introns with abundant antisense transcription**
**1. Correlation between RNAseq densities of each intron and the corresponding exons (Figure**2**B)**	≤ 0	30,141	111	0.009	28,617	1,258	266	5.1 %
**2. Correlation with other introns (Figure**2**C)**	≤ −0.3	8,989	39	0.019	8,529	384	76	5.1 %
3. Intronic density relative to the minimal intronic density in a transcript*** (Figure2**D)**	≥ 10	18,863	104	2.34E-10	17,491	1,101	271	7.3 %
**4. Maximum Intron/Exon Density Ratio (Figure**2**E)**	≥ 1	5,753	95	1.37E-42	4,936	594	223	14.2 %
**5. Coefficient of variation of each intron (Text)**	≤ that of exons	40,948	201	4.31E-16	39,146	1,107	695	4.4 %
**6. DE in any timepoint (Table**3**)**	Presence of a DE bin	25,739	159	1.34E-22	24,626	714	387	4.3 %
**Total number that pass at least one criterion**		82,481	349	4.49E-27	77,611	3,517	1,341	5.9 %
**Maximum number that pass at least two criteria**		35,979	229	1.90E-40	34,315	1,248	416	4.6 %
**Maximum number that pass at least three criteria**		10,111	103	2.02E-30	9,671	319	121	4.4 %

* With unique coordinates.

** Based on 534 Ensembl-specific snoRNAs (see Methods).

*** See Methods.

**** The following was done: 1. Average intronic density was calculated per each time point, introns were sorted based on this value and an intron with minimal value for each time point was identified. 2. Per each time and each intron, the ration of the density for this intron versus the minimal intronic value in this locus was calculated. 3. Introns passing the threshold in at least one timepoint were taken.

In addition, we investigated whether functional non-coding RNAs encoded in introns were derived from the same strand as the gene itself or the opposite strand. Since our cDNA synthesis method does not exclude spurious second-strand cDNA synthesis, we had to account for the fraction of antisense reads that could be derived from this process. We did that by aligning all RNAseq reads to known exon-exon junctions and determining the fraction of exon-exon junction spanning reads thatwere antisense to them. While not all such reads are artificial, for example some of them could be produced by RNA copying [30], this still allowed us to estimate the maximum level of artificial antisense reads present in our dataset. The average fraction of sense reads spanning the splice junctions was found to be 83% and standard deviation 20%. Therefore, introns with a fraction of sense reads ≥63% were considered to be harboring predominantly sense transcripts. It is important to note that such introns could still have antisense transcripts, but the levels of these antisense transcripts would be appreciably lower than the sense transcripts. Similarly, introns with a fraction of sense reads <37% were considered to harbor predominantly antisense transcripts, and those with 37-63% were considered to harbor both sense and anti-sense transcripts (Table 5, Additional file 3: Table S2).

As expected, the majority of introns harbored sense transcripts, with the fraction of introns with evidence of abundant antisense transcripts ranging from 4.3% to 14.2% depending on parameters used. This is significantly less than the previous estimates of 50-70 + % of global antisense transcription obtained in previous genome-wide surveys [31,32], but again it is worth stressing that only abundant antisense transcripts would be detected by this method. Interestingly, the fraction of introns with antisense reads is among the lowest in introns with low variance (low CV) (4.4%) and is the highest in introns with high RNAseq density relative to the corresponding exons (14.2%) (Table 5, Additional file 3: Table S2). This is consistent with what we would expect: antisense transcripts would likely be regulated differently than the sense intronic transcripts and thus the CV of introns with a lot of antisense transcription would be high and they would be excluded by the low CV filter. On the other hand, since antisense transcripts would add to the overall mass of RNA made from introns, the density of RNA signal in such introns would be high and they would be enriched using the second filter.

### Linc RNAs represent a minor fraction of the non-coding transcriptome

Long Intergenic Non-Coding (linc) RNA regions were first identified based on profiling of histone modifications associated with elongating RNA Pol II in mouse [33] and then human tissues [34]. This class of non-coding RNAs has become quite prominent in the past 2–3 years [35] due to in-depth analysis of several linc RNAs, most notably HOTAIR [36].

**Figure 6:**
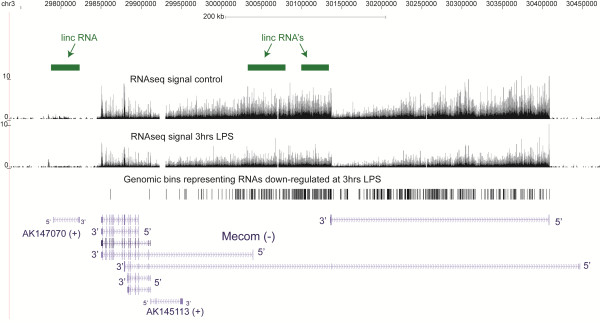
**Examples of lincRNA regions originally found in intergenic regions [**[33]**] that are actually part of longer intronic transcripts.**

We thus investigated the distribution of our informative reads and DE bins among the mouse linc RNA regions published by Guttman et al. In total, we could remap 1,666 out of the original 1,673 regions to mm9. We realized that 530 of those regions originally found in the intergenic space now map to or overlap UCSC Genes as exemplified by Figure 6 where 2 linc RNA regions map to Mecom locus (Figure 6). Interestingly, the genomic span of the observed RNAseq signal in the introns of the Mecom locus is larger than the linc RNA regions (Figure 6), suggesting that when the linc RNA loci overlap the RNAseq signal, they are often shorter than the full extent of the expressed domain. We then sought to determine the abundance of these regions in our RNAseq data based on the fraction of informative reads that map onto genic and intergenic lincs. In total, we found that only ~2.5% of informative reads mapped into genic lincs and only ~0.7% mapped into intergenic lincs (Table 6). This is consistent with the overall notion that the intronic signal typically dominates the transcriptome and lincs that are part of intronic RNAs tend to be more abundant. This is also illustrated by the lincRNA regions in Figure 6: the linc RNA regions on the left outside of the Mecom coding locus are expressed at a much lower level than the two intronic lincs. Also, a minor fraction of DE bins mapped into the linc RNA regions: ~1.1% of non-exonic DE bins mapped to genic linc regions and an additional ~0.9% to intergenic linc regions (Table 6).

**Table 6:** Distribution of informative reads and DE bins in annotated lincRNA regions

**Time point (hours)**	**Informative reads**	Informative reads that overlap withgeniclincRNA regions*	**% of Informative reads that overlap withgeniclincRNA regions**	Informative reads that overlap withintergeniclincRNA regions*	**% of Informative reads that overlap withintergeniclincRNA regions**	Non-exonic DE bins**	Non-exonic DE bins ingeniclinc RNA regions*	% of Non-exonic DE bins ingeniclinc RNA regions*	Non-exonic DE bins inintergeniclinc RNA regions*	% of Non-exonic DE bins inintergeniclinc RNA regions*
0	58,995,885	1,472,090	2.5 %	420,117	0.7 %					
3	67,176,485	1,619,443	2.4 %	482,092	0.7 %	19,433	214	1.1 %	147	0.8 %
6	64,713,929	1,626,306	2.5 %	474,877	0.7 %	8,912	89	1.0 %	78	0.9 %
12	50,425,532	1,268,052	2.5 %	348,418	0.7 %	8,376	84	1.0 %	72	0.9 %
24	52,751,370	1,342,859	2.5 %	387,030	0.7 %	10,128	130	1.3 %	73	0.7 %
48	49,007,599	1,278,355	2.6 %	359,202	0.7 %	8,688	86	1.0 %	65	0.7 %

*As defined by the Additional file 2: Table S1 from Guttman et al., 2009 [33].

**Relative to the control time point.

### Intronic RNAs represent long RNAs

**Figure 7:**
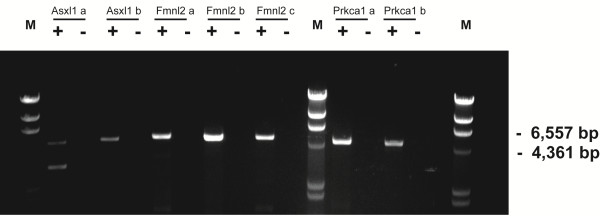
**Detection of long transcripts in intronic regions using RT-PCR.** Reactions were done with (“+”) and without (“-”) reverse transcriptase. The size range where the expected PCR products should fall is shown on the right. M- size standard. See text, Figures 1 &5 and Methods for more details.

It has been reported that cells depleted for the components of RNA degradation machinery, such as the exosome, have higher levels of some non-coding RNAs [37,38]. It has also been reported that stable small RNA products could in fact be detected from longer RNAs [39,40]. It is conceivable that long intronic regions found here were represented mostly by overlapping short RNA molecules that span such intronic regions. As such, we investigated whether intronic transcripts found here could in fact represent long RNA species. To accomplish this, we have selected 3 loci and performed 2–3 RT-PCR experiments per locus with overlapping primer pairs that were designed to amplify products on the order of 4.5-5 kb in length (Figures 1 and 5, Additional file 4 Table S3). In addition, for each RT-PCR experiment, we have used a gene specific primer for reverse transcript located 4-5 kb downstream of the PCR primer pair (Methods). Thus, the entire length of the corresponding RNA from which such RT-PCR product could be obtained would be on the order of 8–10 kb (Additional file 4: Table S3). In fact, we could obtain RT-PCR products of expected lengths for all regions tested (Figure 7). This suggests that the lengths of the intronic RNAs would be larger than 8-10 kb.

## Discussion

The discovery of pervasive transcription of the mammalian genome [5,32,41-43] has provoked intense debate as recently as one or two years ago [6,8,9,44]. With the recent reports by the ENCODE consortium finally setting aside any doubts about wide-spread existence of RNA transcribed from non-coding regions of our genome [11,12], the debate is shifting from the existence of the “dark matter” RNA to its function. Nowhere is this debate sharper than in the intronic regions of protein coding genes that cover ~40% of our genome, and where the majority of non-exonic RNAs map [6,9]. If intronic RNAs, for the most part, simply represent pre-mRNAs or spliced introns en route to degradation, then it would be fair to say that there is likely little functional “dark matter” RNA in the genome. Following similar logic, transcription in the intergenic space would also eventually be populated by exons of the novel transcriptional units separated by presumed non-functional intronic space. This “null hypothesis” could reconcile a view that there is a lot of non-exonic RNA in a cell, while there is little “dark matter” functional RNA. And, while some intronic non-coding RNAs are well known, they could be an exception to the rule.

Here, we challenge this general notion and provide evidence that many intronic RNAs can display features consistent with function: their levels of expression and their biological variation during a physiological time course, or among different individuals of the same strain, can often occur with a magnitude similar to that of exons. Furthermore, levels of intronic RNAs often do not depend on levels of their exonic counterparts. Based on these criteria, many introns produce RNA molecules whose fates in the cell are different from that of exonic RNAs, in response to the important biological stimulus of LPS induced inflammation. The combination of these and other parameters as shown in Table 5 could guide the selection of intronic regions that encode RNAs with functions distinct from thoseof pre-mRNAs, in a given process such as inflammation.

While these arguments do not tell us how intronic RNAs function, they suggest that they should not be ignored or automatically assigned to the category of annotated pre-mRNAs as suggested by van Bakel et al. [8,9]. On the contrary, our results suggest a situation where intronic RNAs and exons have different functional fates in the cell. Overall, we believe that the question of function of intronic RNAs will become key in the genomics of non-coding RNAs and genomics in general. We found a significant fraction of intergenic transcription [10] and in this work approximately 21% of all informative reads and 8% of all DE bins fell within intergenic regions (Table 3). However, as the annotations of the known transcripts expand, most of the intergenic transcript bins will be categorized as intronic or exonic as well. For example, about half of intergenic DE bins overlap ESTs (Table 3), with the majority of them overlapping EST introns (Table 3), suggesting that the labels “intron” or “exon” should not influence an unbiased investigation of function.

This knowledge has an immediate application in molecular diagnostics as intronic RNAs can apparently provide additional information about a biological state that cannot be obtained just by the analysis of exons alone. As a matter of fact, microarray-based analysis of specific biological systems have indeed shown that non-exonic RNAs, including intronic RNAs, not only can be used as diagnostic markers [45,46], they could actually be better discriminators and predictors than exons [47,48]. This is very consistent with the overall conclusions of this work.

This begs the question of what potential functions could be carried out by intronic RNAs. We think that there are at least two likely possibilities: precursors to smaller functional RNAs, and RNA scaffolds. The general theme of production of smaller RNAs from larger precursors is now recognized in the field [49]. For example, miRNAs and other known small RNAs are produced from larger precursors, such as the annotated precursor to mir-21 that overlaps introns of Tmem49 (Figure 4). Among the intronic and intergenic regions upregulated after LPS treatment, there are indeed those overlapping miRNAs (Additional file 5: Figure S2), suggesting that at least some such regions could serve as primary transcripts further processed into the small functional miRNA molecules. It is also interesting in this respect that a cluster of piRNAs was found in the Prkca gene, albeit not in the intron that was found to be highly expressed and changing after LPS induction (Figure 5A). In addition to annotated classes of small RNAs, introns could be processed into yet unknown small RNAs that could have function, potentially similar to that reported previously for Kit RNA degradation products [50]. On the other hand, the role of RNA as a scaffold has been demonstrated by Silver and colleagues [51]. Considering the very large size of some of the intronic regions exemplified by the Prkca and Slc24a3 genes (Figure 5 and Additional file 2: Table S1), it is an attractive possibility that these molecules serve as massive scaffolds for various protein-binding factors that could even bridge distal DNA loci in the 3-dimensional volume of the nucleus [15].

While the fact of pervasive transcription is firmly established now as mentioned above, as history shows, it can fade somewhat with time, with focus instead shifting to separately developed lists of long non-coding RNAs detected in specific experiments or filtered by certain properties that hint at functionality [33,34,52-55]. In this respect, it is important to realize that these lists of non-coding RNAs usually only cover a few percent of the genome, representing only a small fraction of the original pervasiveness of transcription. And, as we have shown in this work with the list of the lincRNA regions [33], the contribution of such lists to the overall mass of cellular RNA is small and it’s not uncommon for such regions to represent longer transcripts (Figure 6).

## Conclusions

This study presents a highly quantitative, comprehensive and unbiased RNAseq dataset showing that most of the RNAs that change during inflammation correspond to the non-coding, predominately intronic, regions of the genome. These non-coding RNAs would not have been detected if only the exons of protein-coding mRNAs or relatively few non-coding RNAs from existing lists were considered as most of the genomic regions they are derived from are not annotated. In general, large numbers of intronic RNAs have properties comparable to those of stand-alone functional RNA species, suggesting that they are more than just discardable parts of pre-mRNAs. In summary, these results argue for a global change in thinking away from one where intronic RNAs are automatically relegated into the pre-mRNA category. Rather, the community should analyze RNAs from these regions with the same interest and rigor as it does mRNAs or linc RNAs.

## Methods

### Biological material

Female Balb/c mice, approx. 6–8 weeks old (Balb/c:OlaHsd, nulliparous and non-pregnant) were used in this study as a well-established model of LPS-induced inflammation. All animals were weighed and randomized prior to their first challenge: in consideration of their weight they were distributed evenly to groups of eight animals each. For the induction of respiratory inflammation, all animals were exposed to a defined LPS aerosol, except the negative control group which was exposed to clean air only. The aerosols were generated using an Aeroneb nebulizer. The LPS inhalation was done using aerosolized lipopolysaccharide (deposited dose approx. 20 ng LPS) with 0.021% LPS solution for an inhalation period of 10 min on three consecutive days. After the final challenge a necropsy was performed at the following time points: 0 (clear air only, no LPS), 3, 6, 12, 24 and 48 hours.

Animals were treated using the vehicle 7 days before first inhalative challenge and daily 1 hour before challenge to LPS or clean air (the control group). Animals from all groups were sacrificed painlessly with an overdose of pentobarbital sodium (Narcoren®) 0 (control) and 3, 6, 12, 24 and 48 hours after LPS-challenge. Seven animals were assessed per time point. The inflammatory status in lungs was analysed including the numbers of macrophages/monocytes, neutrophils, eosinophils and lymphocytes by counting a total number of 300 cells per cytospot.

Whole lungs were excised, cut into small pieces (max 0.5 cm diameter) and transferred in a sufficient volume of RNAlater (Ambion) and frozen in liquid nitrogen. The tissue samples were stored at −70°C until RNA isolation.

### Isolation of total RNA from tissues

Total RNA from tissue samples and cell lines was extracted using TRIzol® reagent (Invitrogen Corp. Catalog No. 15596–026) using manufacturer’s protocol. The sample was homogenized while suspended within an appropriate volume (10x sample volume) of reagent. After a brief incubation at room temperature, the samples were centrifuged to remove insoluble material and the supernatant transferred to a fresh tube. Choloroform (0.2x TRIzol volume) was then added to the contents of the new tube and vigorously vortexed. After a few minutes of incubation at room temperature samples were then centrifuged for 15 minutes at 12000 x g at 4°C. This results in the formation of three distinct phases. The topmost aqueous phase, which contains RNA, was then carefully transferred to a new tube. The addition of isopropyl alcohol (0.5X TRIzol volume) to the samples followed by a 10 minute room temperature incubation and a subsequent 10 minute centrifugation at 12000 x g at 4°C precipitated the RNA into a gel-like pellet at the bottom of the tube. After the removal of the supernatant, the pellet was washed twice in 1 ml of ice-cold 75% ethanol. The resultant pellet of RNA was then allowed to dry for about 5–10 minutes and finally resuspended in DEPC-treated water. The quality and the quantity of the resulting RNA was then measured using spectrophotometric techniques on a NanoDrop instrument (Thermo Scientific).

### RNA processing and SMS

Total RNA was first DNase treated to remove any residual DNA. Approximately 40 μg of total RNA (with 20 μl 10x buffer, 4 μl DNase 1 (Ambion, AM8170G), 2 μl Rnase-out (Invitrogen, 10777019) in a total volume of 200ul) is incubated for 30 minutes at 37°C. Samples are then cleaned using the RNeasy MinElute cleanup kit (Qiagen, 74204) following manufacturer’s protocol. In brief, 700 μl Buffer RLT and 500 μl 100% Ethanol are added to the sample which is then added to a MinElute spin column. The columns are washed with 500ul Buffer RPE followed by 500 μl 80% Ethanol. After an additional 2 minute centrifugation to remove any residual ethanol the sample is eluted in 14ul DEPC treated water. Quality of RNA was then assessed using an Agilent 2100 Bioanalyzer and their RNA 6000 Nano Kit (Agilent, 5067–1511) using manufacturer's protocol and the sample was quantified using a Nanodrop as per above. Samples were depleted for rRNA through the use of the RiboMinus Eukaryote Kit for RNA-Seq (Invitrogen, A10837-08) following the manufacturer's protocol. The RNA was then converted into cDNA, and subjected to SMS as previously described [10]. Processing of SMS reads, alignments to the genome and data analysis was done as previously described [10]. The reads were trimmed to a minimal length of 25 bases resulting in an average size of 35 bases and maximum of 71 bases and aligned with a minimal normalized score = 4.5 [10].

All genomic coordinates listed throughout this work correspond to the mm9 version of the mouse genome.

### Intronic intervals preparation

We extracted intronic intervals from the knownGene.txt file downloaded from the UCSC genome browser site, based on the mm9 version of the mouse genome and last modified 30-May-2011 00:13. We then removed parts of introns that overlapped exons annotated in the same knownGene.txt file, also see Additional file 1: Figure S1. Random chromosomes were ignored. In total, 198,248 unique intronic intervals were finally extracted.

### Calculation of RNA densities in introns and exons

To calculate intronic densities, we counted number of reads that fall within each intronic interval (see Additional file 1: Figure S1), normalized this number by 10 M informative reads and then calculated the density of reads per 1 kb for each intronic interval. Since our intronic intervals do not overlap UCSC exons, reads originated from that exons are not counted in intronic densities. A read that overlap exons and introns was counted as 0.5 read in both exonic and intronic counts. We also calculated normalized exon densities for the entire length of each annotated transcript that harbors each intron. We then paired the values of the intronic and exonic densities for each animal for each time point separately.

### Calculation of global correlation between levels of introns and their corresponding exons

This analysis encompassed 197,631 unique introns longer than 30 nt in 47,773 transcripts annotated in the UCSC Genes database [23]. In total, for each animal we generated 459,132 pairs of intron-exon values x 7 animals x 6 time points (“Total Intron-Exon Time-Course Dataset”), with one intron on average being present in 2.3 transcripts. We then combined the data for all animals/time-point (7 animals x 6 time points), removed data points where both exonic and intronic densities were equal to zero, and then sorted the data by exonic density and picked the top half of the data points, in order to remove transcripts with low read counts. The minimal exonic density in this dataset was 30.88 (per 10 M mapped reads per 1 kb of exonic sequence) which translated into a minimum of 7.6 actual reads per 1 kb in the sample with the smallest number of reads. Overall, this filtered dataset yielded 8,918,127 total data points of exon-intron densities, we will refer to this dataset as the “Total Intron-Exon Pair-Wise Dataset”. This dataset was used to generate the plot in the Figure 2A.

### Calculation of correlation between individual introns and their corresponding exons throughout the time course

We started with the “Total Intron-Exon Time-Course Dataset”, removed the intron-exon combinations with zero reads in all exonic or intronic samples and then selected the top half exonic expressors based on average density of exonic signal to generate the “Top expressed Intron-Exon Time-Course Dataset”. This dataset was used to generate the correlations plotted in the Figure 2B.

### Calculation of intron-intron correlation within the same locus

We took all loci used to generate the histogram in the Figure 2B. Since that dataset was selected based on the exonic expression, we wanted to remove any loci with low intronic signal to make sure that any low correlation is not due to stochastic variation in signal. For each locus we calculated maximum intronic density in any of the 42 animals, then we kept only those introns whose locus fell in the top half of maximum intronic density. We then calculated for every intron of a transcript with 2 or more introns a Spearman correlation of its RNA levels with those of the other introns in all 42 animals. In total, we used 59,027 unique introns for this analysis. The results of this analysis are shown in the Figure 2C.

### Calculation of coefficient of variation of introns and corresponding exons

We started with a total of 2,754,792 intron-exon combinations (459,132 intron-exon combinations x 6 time points) to generate the “Total Intron-Exon Per Time-Point Dataset”. One complication that we faced was that the CV of a transcript level among animals depends on the expression level of a transcript, with more abundant transcripts having less variation – as would be expected, since more abundant transcripts could be measured more reliably than the less abundant ones. Indeed, the Spearman rank correlation between average RNA signal density and the corresponding CV was −0.58, indicating that transcripts with lower abundance have higher CV. Since introns had a tendency to have lower abundance than exons, their variation would be higher due to the intrinsically higher variation of detection of lower-abundant RNAs. To avoid intronic transcripts with low levels of expression in this experiment, we sorted the “Total Intron-Exon Per Time-Point Dataset” by average intronic density and selected the top half of data points for analysis where average exonic density was positive (1,231,535 intron-exon combinations). The median CV for introns and exons in the resulting dataset were 35.37% and 18.97% respectively and the median density of an intron being 18.16% of the corresponding exons.

### Analysis of differential expression using the genomic bins approach

Optimal detection of regions of transcription requires the use of nested bins of different sizes. For example, exons of protein-coding mRNAs that have a median size on the order of 100 bp in the mouse genome would be expected to be optimally detected with a smaller size bin on the order of 100 bp. On the other hand, relatively low abundance longer transcripts would be detected best with a bin of longer size that has a chance to capture more SMS reads representing such transcripts. Therefore, genome sequence was split into non-overlapping bins of variable size 100, 200, 500 and 1000 bp and expression density of each bin for each animal for each time point was calculated. Up-regulated and down-regulated bins (fold change > 2 between densities averaged across 7 animals) on each time point comparing to control (which is zero time point) were identified. One-tailed paired Student’s *t*-test was used to estimate the fold change significance across 7 animals and p-value < 0.001 cutoff was applied. If one group consists only of zero values, then artificially 0.5 read is added to one animal and 0.5 read is subtracted from another animal of the group before expression density and Student score calculation.

Bins of different sizes were then merged together. Whenever a bin of smaller size overlapped a bin of a larger size, a bin of the smaller size was chosen as it is more likely to represent the accurate bounds of the detected transcribed element. The power of a more precise bin approach is illustrated by detection of a specific isoform of Adora 3 consisting of only two exons out of a total of 9 known for this locus and upregulated at the 3 hour time point (Additional file 6: Figure S3).

To account for multiple testing, we randomly shuffled within each animal the expression values of 6.2 M of 100 bp bins that had non-zero expression at least for one animal at one time point. The number of bins that had p-value cutoff below 0.001 after the shuffling was at least 100 times less than the number of such bins before the shuffling. This control test allows us to infer that the false discovery rate of our statistical method that extracts DE bins is below 1%.

### Identification of transcripts that contain differentially expressed intronic bins with no change in exons

Genomic bins located in introns (overlap with exons not allowed) of annotated UCSC Genes were selected from list of up-regulated and down-regulated on 3 hrs after LPS bins described above. Up-regulated or down-regulated bins where the corresponding gene had respectively at least one up-regulated or down-regulated exon at 3 hrs after LPS (fold change > 1.414, p-value < 0.01) were filtered out from the list.

### Calculation of overlap with Ensembl-specific snoRNAs

1,383 snoRNAs annotated by the Ensembl and not UCSC Genes database were selected to assess enrichment of functional non-coding RNAs presented in Table 5. Of the 1,383 Ensemble-specific snoRNAs, 534 were located in 198,248 unique introns used in this paper, specifically in 528 introns, and became the test set for the enrichment of functional RNAs in the introns. Tested introns dataset was different for each of 6 types of analysis presented in Table 5 as described in the text. Subset of introns was selected from tested introns dataset using criteria presented in Table 5. Numbers of introns overlapping the 534 snoRNAs from each tested dataset was found. P-value was calculated using Fisher exact test, tail of hypergeometric distribution was calculated

$$\;p-value=\sum_{i=m}^{n}\frac{\left(\begin{array}{c} n\\ i \end{array}\right)\left(\begin{array}{c} N-n\\ M-i \end{array}\right)}{\left(\begin{array}{c} N\\ M \end{array}\right)}\text{,}$$

where

N - number of unique Introns in the tested dataset,

M - number of unique Introns from tested dataset passing the threshold,

n - number of unique Introns from N overlapping snoRNAs,

m - number of unique Introns from M overlapping snoRNAs.

### RT-PCR analysis

First strand synthesis was performed using Superscript III (Invitrogen, 18080–051) following the manufacturer’s protocols with 200 ng Total DNase-free RNA (see above) as a template. See the Additional file 4: Table S3 for Gene specific primers used. Each sample had RNA removed with the addition of 1ul Rnase H, incubated at 37°C for 30 minutes. Subsequent PCR was performed using Long Amp Taq PCR Kit (NEB, E5200S) following the manufacturer’s protocol on 2.5ul cDNA in a 25ul final volume (94°C 30s, 34x (94°C 30 s, 55°C 30s, 65°C 5 m), 65°C 10 m). See the Additional file 4: Table S3 for PCR primers used. Products were run on 1% agarose gel.

## Competing interests

Authors declare no competing interests.

## Authors’ contributions

DS, TE and PK performed the statistical and bioinformatics analyses; MT and ZY performed sample preparation and SMS sequencing; CW and SUI provided theoretical insights; GstL, BS and TAM designed and performed the inflammation time course study; PK and GstL provided the theoretical framework and guidance for the intronic RNA analysis and wrote the manuscript. All authors read and approved the final manuscript.

## Supplementary Material

Additional file 1 **Figure S1.** A scheme of the strategy to partition intronic coordinates in the cases of overlapping transcripts. Boxes –exons, lines – introns. Regions 1–4 were used to calculate the average read density of the corresponding introns. Click here for file

Additional file 2 **Table S1.** Correlation of specified introns of Prkca and Slc24a3 with their corresponding exons. Click here for file

Additional file 3 **Table S2.** Properties of individual mouse introns. Click here for file

Additional file 4 **Table S3.** Details of RT-PCR analysis in selected mouse introns. Click here for file

Additional file 5 **Figure S2.** An example of DE bins specifically detecting a specific up-regulated isoform of Adora3 locus. Click here for file

Additional file 6 **Figure S3.** Examples of DE bins detecting regions around annotated miRNAs, found both in an intron (mir-135b, A) and an interegenic region (mir-146a, B). Click here for file
