# Supplementary material for: Intronic RNAs constitute the major fraction of the non-coding RNA in mammalian cells
Source: BMC Genomics. 2012 Sep 24;13:504. doi: 10.1186/1471-2164-13-504 (PMC3507791; doi:10.1186/1471-2164-13-504)
Supplement: Additional file 1 — Figure S1. A scheme of the strategy to partition intronic coordinates in the cases of overlapping transcripts. Boxes –exons, lines – introns. Regions 1–4 were used to calculate the average read density of the corresponding introns. [file 1471-2164-13-504-S1.pdf]

Strategy for calculating intronic densities in overlapping transcripts

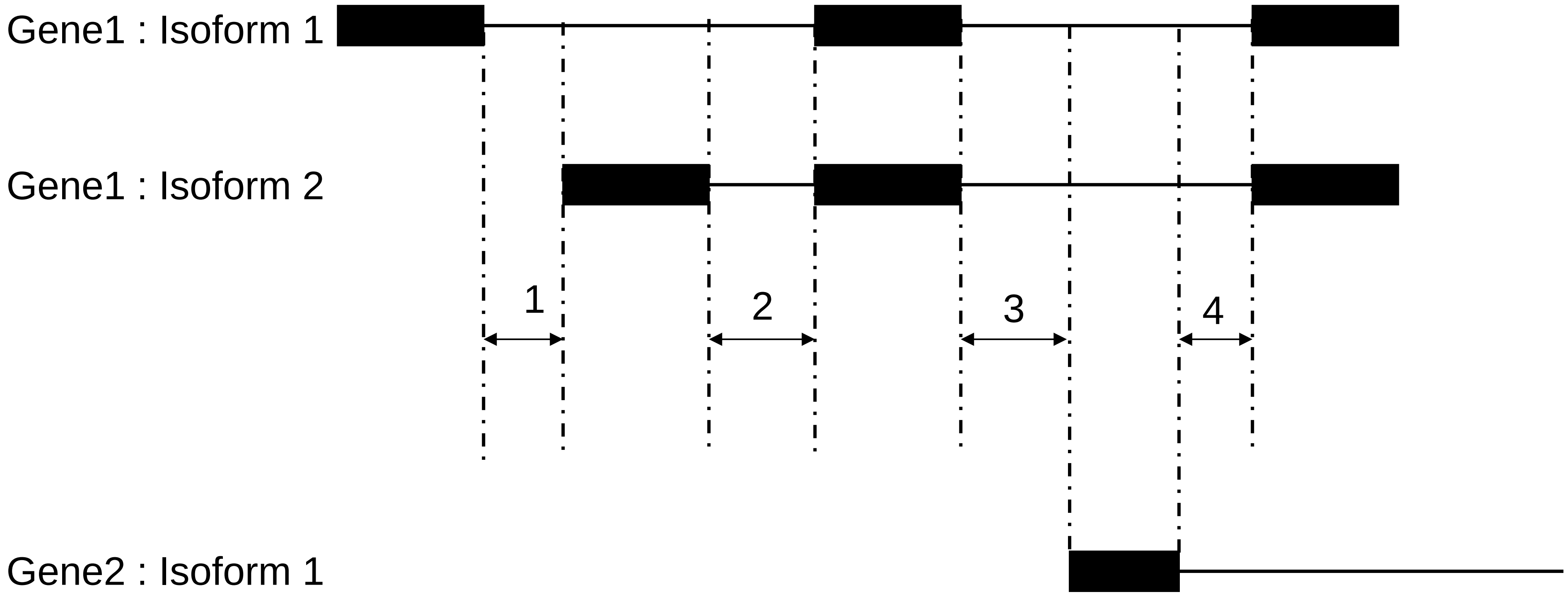

Intronic densities were calculated based on the coordinates of regions 1-4.

Figure S1
