# Supplementary figures and images for: Intronic RNAs constitute the major fraction of the non-coding RNA in mammalian cells
Source: BMC Genomics. 2012 Sep 24;13:504. doi: 10.1186/1471-2164-13-504 (PMC3507791; doi:10.1186/1471-2164-13-504)

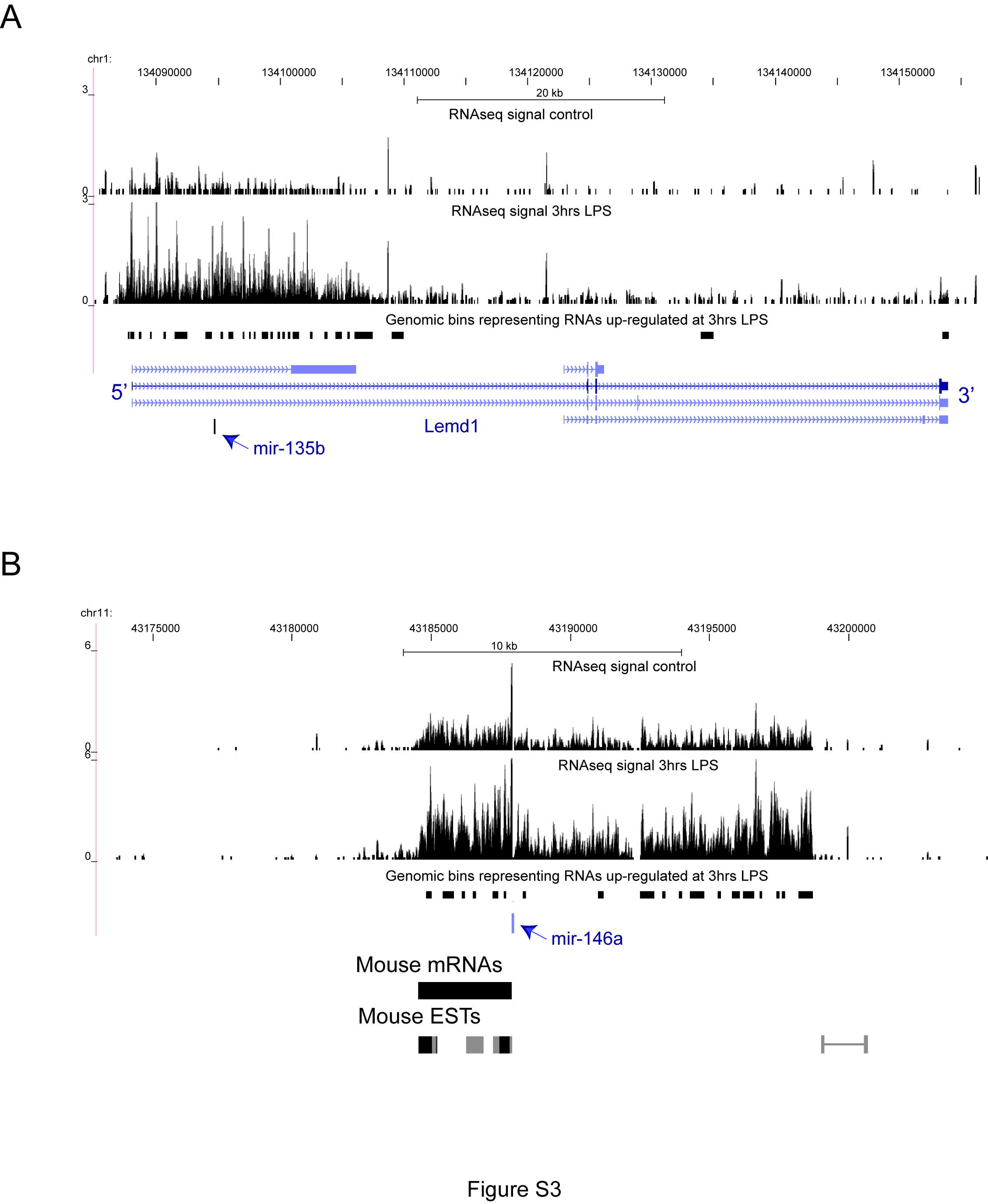

Supplement: Additional file 5 — Figure S2. An example of DE bins specifically detecting a specific up-regulated isoform of Adora3 locus. [file 1471-2164-13-504-S5.tiff]

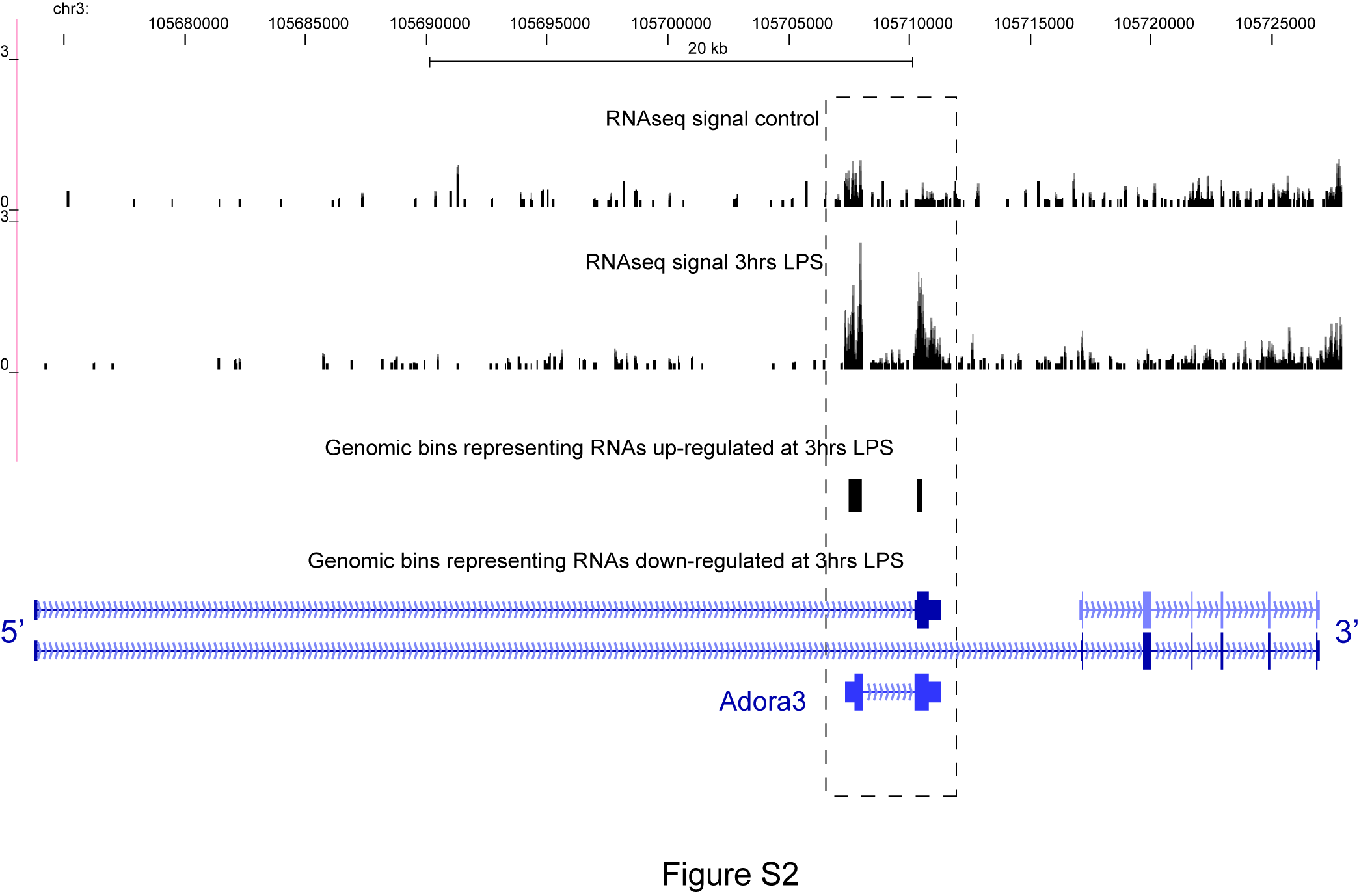

Supplement: Additional file 6 — Figure S3. Examples of DE bins detecting regions around annotated miRNAs, found both in an intron (mir-135b, A) and an interegenic region (mir-146a, B). [file 1471-2164-13-504-S6.tiff]
